# Identifying studies examining the validity of instruments for use as outcome measures in child and adolescent forensic mental health services: a systematic review

**DOI:** 10.1007/s00787-024-02514-7

**Published:** 2024-08-07

**Authors:** Graham Walker, Naomi Wilson, Clare S. Allely, Allan Thomson, Helen Smith, Jason Lang

**Affiliations:** 1 University of Glasgow, Scotland, UK; 2 NHS Scotland, Scotland, UK; 3 University of Salford, England, UK; 4 NHS Ayrshire & Arran, Scotland, UK

**Keywords:** Outcome, Instrument, Forensic, Children, Adolescent

## Abstract

**Background:**

Outcome measurement in child and adolescent forensic mental health services can support service improvement, research, and patient progress evaluation. This systematic review aimed to identify studies which validate structured instruments available for use as outcome measures in the child and adolescent forensic mental health service cohort and assess the quality of these studies.

**Methods:**

A systematic review was conducted following PRISMA guidelines. Studies were identified by searching six online databases in November 2023. The quality and risk of bias of each study meeting inclusion criteria was independently assessed by two authors using the Crowe Critical Appraisal Tool. Results were synthesised narratively.

**Results:**

A total of eight studies were identified which met inclusion criteria. These looked at six instruments which primarily focused on outcome measures in the areas of treatment motivation, level of functioning, psychiatric symptoms, care needs and response to social situations. Papers scored between 17/40 and 30/40 on the Crowe Critical Appraisal Tool. Studies were rated as low (*n* = 1), moderate (*n* = 6), high (*n* = 1) or very high quality (*n* = 0).

**Conclusions:**

Despite the large number of structured instruments potentially available, evidence for their use as outcome measures in child and adolescent forensic mental health services is limited. Future research should aim to validate current structured instruments for use in the forensic child and adolescent setting, with consideration of whether new instruments should be developed specifically for this group. Such instruments should be developed with both young people as service users and professionals who will be utilising the instrument in mind.

**Supplementary Information:**

The online version contains supplementary material available at 10.1007/s00787-024-02514-7.

## Introduction

Structured instruments are commonly used in the forensic mental healthcare setting however evidence for their use as outcome measures mostly focus on risk [1]. Such tools serve multiple purposes including to measure response to treatment, and help identify individuals personal, and more broadly service, needs. Such tools may also shape quality improvement and inform risk assessment [2]. Such instruments tend to be reasonably simple to perform by professional staff who have undergone appropriate short-term training and can give clear results for research and quality improvement purposes. Despite this, they can have a specified area of focus and therefore have the potential to miss important information about a service user or clinical outcome [2]. Some clinicians have raised concerns about the value of these instruments, the possibility of misuse of data, poor technological support, and the additional workload demands [3].

A subset of forensic mental health clients with particularly complex needs are those cared for by forensic child and adolescent services [4]. Approximately 3500 young people under 18 are admitted to inpatient Child and Adolescent Mental Health (CAMHS) units across the United Kingdom (UK) each year [5]. Globally, recent data indicates that there has been an increase in the prevalence of mental health disorders and neurodivergence being diagnosed in children and adolescents, including depression, bipolar disorder, attention-deficit/hyperactivity disorder, and autism [6, 7]. Youth who are incarcerated in secure detention and commitment settings display a complex array of educational, behavioural, and mental health issues that affect the services they require, as well as their responsiveness to interventions [8, 9].

NHS England and the Royal College of Psychiatrists both continue to emphasise the importance of routine outcome measurement in psychiatry in the United Kingdom [2]. Across Europe, including in Gemany and the Netherlands, the importance of routine outcome measurement in mental healthcare has also continued to be highlighted [10, 11]. An international consensus has been set for standard outcome measures in the general CAMHS population, including for those who experience anxiety, depression, obsessive-compulsive disorder, and post-traumatic stress disorder [12]. A report by the Department of Health concluded that there should be agreed national minimum standards for community forensic child and adolescent mental health services (FCAMHS) and a standard commissioning framework to provide a level of national consistency in provision [13]. Since the report was published in 2013 there has not been a further update on such minimum standards. Despite this, resources within the FCAMHS system continue to grow. The first National Secure Adolescent Inpatient Service for Scotland (Foxgrove) has recently been established. This is an eight bed medium secure adolescent mental health facility [14]. In such units, ongoing use of appropriate structured instruments as outcome measures will be implemented.

Ideally, any structured instrument being used as an outcome measure would be appropriately validated for use within the given population [2]. Despite the large number of instruments potentially available, evidence for their use as outcome measures in adult forensic mental health services is limited [15]. Historically, outcome measures have either been targeted towards adults or children and adolescents, reflecting the traditional demarcations within the mental health care system. Suitable outcome measures are required that are appropriate to a persons developmental, social and emotional stages [16].

The Child Outcomes Research Consortium (CORC) maintains an online resource detailing appropriate references to the psychometric properties (reliability, test-retest reliability, concurrent validity, discriminant validity) of each outcome and experience measuring tool, relevant for use within child and adolescent psychiatry settings [17]. There is no tool listed with evidence of psychometric property testing in a forensic CAMHS cohort [17]. However, there is a range of validated measurement tools for monitoring progress and outcomes for children and young people in contact with generic CAMHS services [17].

These generic measures may not all be as useful in measuring outcomes in the young people seen by an FCAMHS team and it may be that further appraisal of this aspect of clinical outcome monitoring should be reconsidered in the light of the complex needs of this population [13, 18]. The authors believe that outcome measures which have been validated for use within the general child and adolescent psychiatric population are not nessesarily sufficient for use in the FCAMHS population, where specific validation is required. Service users cared for within the forensic psychiatric system are more likely to represent a risk of harm to themselves and others [4]. Accordingly, they may have longer periods of treatment under more restrictive conditions. They may be more likely to be transferred between varying settings such as custodial, hospital and secure community environments [19]. Such individuals have their own distinct perception of what defines their recovery- an important element of a number of outcome measures [19]. A specific example of an issue with using a generic CAMHS outcome measure (the HoNOSCA) for use in the FCAMHS population is raised below, where raters struggled to score as accurately on this tool due to the more extreme nature of presentations, as well as notable differences around family life, relationships and school attendance. In this study, specific modifications were suggested for use of the HoNOSCA in the FCAMHS population [20].

Previous systematic reviews have looked at structured instruments as outcome measures in an adult forensic population [15] and general CAMHS population [16, 21, 22]. In 2020 Koh et al. conducted a systematic review exploring youth violence risk measures but did not include clinical outcome measures in their analysis [23]. We were unable to find wider systematic reviews studying wider validation of structured instruments as outcome measures in the child and adolescent forensic mental health service population. This is the first systematic review to identify studies which validate structured instruments available for use as outcome measures in the child and adolescent forensic mental health service cohort and examine the quality of these studies.

## Methods

This review followed the Preferred Reporting Items for Systematic Reviews and Meta-Analyses (PRISMA) guidelines [24]. Our review protocol was prospectively registered on PROSPERO (registration number CRD42024495300), with feedback on the protocol incorporated into the final study design.

### Eligibility criteria

Included papers described validation of relevant structured instruments being used as outcome measures in FCAMHS (including inpatient secure services, secure care, prison and community forensic services). Included studies had a proportion of the cohort aged under 18, who were cared for within an FCAMHS setting. Papers were written in English language and published in a peer reviewed journal.

Studies which focused specifically on non-forensic mental health settings were excluded. Instruments which solely focused on risk were excluded as we aimed to focus on outcomes measuring the progress and needs of the individual service user. Papers describing assessments of personality traits or intelligence (which are generally not dynamic) and competency to stand trial or malingering (which are outcomes related to the legal process rather than treatment response) were excluded. We have excluded conference abstracts, book chapters, review articles and other non-peer-reviewed literature. Table 1 summarises our inclusion/ exclusion criteria in population, intervention, comparison and outcomes (PICO) format.

**Table 1:** Inclusion/ exclusion criteria in PICO format

PICO Component	Inclusion Criteria	Exclusion Criteria
Population	Proportion of cohort aged under 18	Full cohort aged over 18
Intervention	Cohort cared for within FCAMHS setting (including low to high security inpatient, secure care, prison, outpatient settings)	Non-forensic mental health settings (including young people with forensic history in generic CAMHS setting)
Outcome	Outcomes measuring progress and needs of individual service user	Outcome focusing on risk, personality traits or intelligence. Outcome focusing on competency to stand trial or malingering.
Publication	Paper written in English language Published in peer reviewed journal	Paper not translated into English Non-peer-reviewed literature
Study Design	Validation studies	Non-validation studies (e.g. case series and qualitative studies)

Table 1 shows a summary of our inclusion/ exclusion criteria in PICO format, broken down by population, intervention, outcome, publication and study design

### Information sources

To identify relevant studies, we searched six online databases; MEDLINE, EMBASE, PsycINFO, CINAHL, Web of Science Core Collection and Cochrane Library, running our search on 8 November 2023. We reviewed the reference lists of all included papers. The quality of evidence for psychometric properties and risk of bias was summarised for each instrument using the Crowe Critical Appraisal Tool (CCAT) [25]. The CCAT is a well validated tool that it is adaptable across a wide range of research designs, and has widespread acceptance [26–28]. The authors were significantly familiarised with its use.

### Search strategy

Studies on measurement properties are sometimes difficult to find in PubMed or other databases due to poor indexing, large variation in terminology, and poor reporting of measurement properties. Therefore, COSMIN have developed two validated search filters for finding studies on measurement properties in PubMed, based on primary research by Terwee 2009 [29]. The COSMIN filter has been translated for validation in other databases, however this has not been validated [30]. Despite this, during scoping searches a professional librarian and one author (GW) reviewed the generated results for MEDLINE, EMBASE, CINAHL and Web of Science and found them to be appropriate to the aims of the systematic review. Translations are not provided for PsycINFO via OVID or Cochrane Library and therefore we have generated our own translations for these, considering Terwee’s original validated search strategy [29].

The following search strategy was used across the six identified databases. In summary, we combined the following searches using key words in combination with direct searching for relevant terms:

### Step 1

Search 1: Child and adolescent cohort.

Search 2: Forensic/ secure cohort.

Search 3: Mental illness or Neurodevelopmental disorder.

Search 4: COSMIN filter for measuring properties of measuring instruments.

### Step 2

Search 1: Child and adolescent cohort **AND** Search 2: Forensic/ secure cohort **AND** Search 3: Mental illness or Neurodevelopmental disorder **AND** Search 4: COSMIN filter for measuring properties of measuring instruments **NOT***exclusion criteria*.

Our full search strategy is detailed in appendix 1.

### Study records

#### Data management

Records were managed through EndNote 21, a specific software for managing bibliographies [31]. A standardised data collection tool was utilised to extract data and quality ratings were added for each paper using the CCAT [25].

### Selection process

**Fig. 1:**
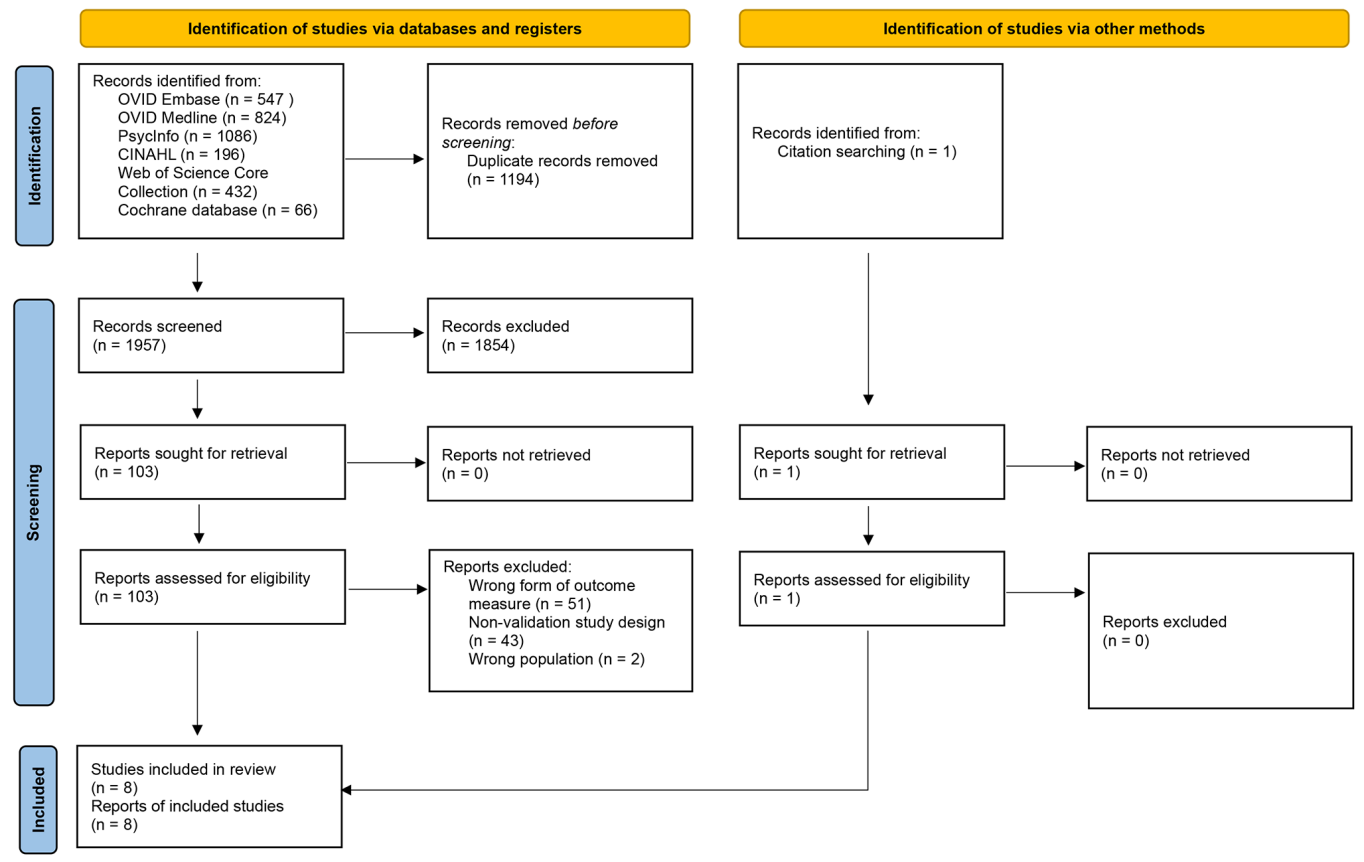
PRISMA Flow Diagram

Titles and abstracts of studies retrieved using the search strategy were independently screened by two reviewing authors (GW and NW) to identify studies that met the inclusion criteria outlined above. Full text was obtained and again independently reviewed by two authors (GW and NW) for papers which were deemed to be potentially relevant to our findings on title and abstract screening (see Fig. 1). In case of any disagreement around meeting inclusion criteria, two authors discussed such papers, and further discussion was held with a third author (JL), as required.

### Data collection process

Relevant data from included papers (such as patient demographics, setting and outcome measurement tools used) were extracted, and plotted in the data collection tool.

### Data items

For included studies, 2 authors (GW and MW) independently extracted data on sample size, mean age of population, sex, setting, participants, country of origin, outcome measurement tool used, and the conclusion of the study on validation for use of the outcome measure in an FCAMHS cohort. Both authors subsequently merged the extracted results into Table 2. Once we included all relevant articles, we subsequently rated each for quality and bias using the CCAT.

### Rating of quality and risk of bias in individual studies

Using the CCAT, we critically appraised each included study (at study level) across eight domains and scored for quality out of 40. Using these ratings, papers were allocated to one of four categories, ranging from very high quality (VHQ) papers (score of 35 or greater), high-quality (HQ) papers (score of 30 or greater), moderate-quality (MQ) papers (score 20 or greater) or low-quality (LQ) papers (score of less than 20).

The CCAT assesses for risk of bias in two of its categories; design (with item descriptors of potential bias, confounding variables, effect modifiers, interactions, sequence generation, group allocation, group balance (and by whom), equivalent treatment of participants/cases/groups) and discussion (interpretation; account for bias, confounding/effect modifiers/interactions/imprecision). Independently from CCAT scoring, we have considered potential sources of authorship bias, in the form of both financial and non-financial conflict of interest.

### Data synthesis

We provide a narrative synthesis of the findings from the included studies as detailed above. There was not scope for meta-analysis because of the range of different outcomes measured across the small number of existing trials.

### Confidence in cumulative evidence

Two authors (GW and NW) used the CCAT to rate the quality of each included study, and in case of any disagreement in score, the opinion of a third author (JL) was conferenced.

## Results

### Search outcome

After applying the search protocol described above to the six databases and omitting duplicates, 1957 articles were returned. Following review of title and abstract of these articles, 1854 articles were excluded as they did not meet the inclusion criteria (see Fig. 1). One further paper was found via screening of abstract references of included papers. In total, 104 articles proceeded to full text review. Subsequently 96 further articles were found not to meet the inclusion criteria. In total, eight articles were rated for quality and data extracted.

Included papers are summarised in Table 2. Mean age of participants ranged from 14 to 20.26. More participants were male in all except for one study. Three studies took place predominantly in an inpatient forensic psychiatric unit, three studies took place in a secure youth care setting and the other two studies took place in a youth prison and community setting respectively. Studies took place in England (*n* = 3), the Netherlands (*n* = 4) and Germany (*n* = 1). One study looked at participants aged 17 to 26 (median age 20.26) [32]. Another study looked at a range of service users in general adolescent (*n* = 18/40) and forensic psychiatric services (*n* = 22/40) [33].

The eight articles which met inclusion criteria looked at six instruments which primarily focused on outcome measures in the areas of treatment motivation, level of functioning, psychiatric symptoms, care needs and response to social situations. These six instruments identified were the BARO (Dutch: BAsisRaadsOnderzoek), ATMQ (The Adolescent Treatment Motivation Questionnaire), HoNOSCA (Health of the Nation Outcome Scales for Children and Adolescents), S.NASA (Salford Needs Assessment Schedule for Adolescents), TOPS-A (Taxonomy of Problematic Social Situations-Adolescent self-report version) and FIOS (Forensic Inpatient Observation Scale). Two included articles each validated the ATMQ and HoNOSCA, with one included article validating the four remaining outcome measures.

**Table 2:** Summary of included studies

	Population			Instrument administration				
Reference	*N*	Age – mean (SD)	Sex, male *n* (%)	Setting	Participants	Country	Version	Conclusion of study on validation for use in FCAMHS cohort
Doreleijers et al., 2011 [34]	295	15.9 (1.8)	268 (91)	Community	Juvenile Offenders	Netherlands	BARO	• Face validity: Not assessed. • Construct Validity: Not assessed. • Internal consistency: Cronbachs alpha was either good (0.88) or sufficient (0.70) • Concurrent Validity: Strong [*r* = 0.69; *p* < 0.001]. • Discriminatory Validity: Measured by the area under curve and was determined to be strong [0.81 (95% CI: 0.69–0.93, *p* < 0.0001)]. • Inter-rater Reliability: Not assessed. • Test-retest reliability: Not assessed. • Perceived Usefulness: Majority of social workers administering the tool rated its usefulness as ‘good’ or ‘very good’ (79.4%).
Heynen et al., 2017 [32]	76	20.3 (2.2)	76 (100)	German Youth Prison	Adolescent and young adult prisoners	Germany	ATMQ	• Face Validity: Not assessed. • Construct Validity: 8 item model showed a good fit to the data, indicating construct validity of the German ATMQ: RMSEA = 0.000, CFI = 0.928, NFI = 0.992, TLI = 0.899, χ2 (20) = 29.797, *p* = 0.073. • Internal Consistency: Cronbach’s Alpha was good (α = 0.79). • Concurrent Validity: ATMQ items strongly correlated with related items on the Prison Group Climate Instrument suggesting good concurrent validity. • Discriminatory Validity: Not assessed. • Inter-rater Reliability: Not assessed. • Test-retest reliability: Not assessed. • Perceived Usefulness: Not assessed.
Hunt et al., 2009 [20]	24	16.6	8 (33.3)	Secure Inpatient Adolescent Unit	Juvenile Forensic Inpatients	England	HoNOSCA	• Face Validity: Not assessed. • Construct Validity: Not assessed. • Internal Consistency: Not assessed. • Concurrent Validity: Adequately correlated with relation to the Brief Psychiatric Rating Scale, the Beck Depression Inventory and the Global Assessment Scale/Children’s Global Assessment Scale. • Discriminatory Validity: Not assessed. • Inter-rater Reliability: Raters total scores were significantly correlated (*r* = 0.92, *p* < 0.01). • Test-retest reliability: Not assessed. • Perceived Usefulness: Not assessed.
Kroll et al., 1999 [33]	40	15.5 (1.5)	25 (62.5)	Secure units, psychiatric inpatient units, and adolescent forensic services	Psychiatric Inpatients	England	S.NASA	• Face Validity: Reported as ‘good’ • Construct Validity: Not assessed. • Internal Consistency: Not assessed. • Concurrent Validity: Not assessed. • Discriminatory Validity: Not assessed. • Inter-rater Reliability: Kappa coefficients should good inter-rater reliability for both categorical items and ordinal scales. • Test–retest reliability: Intra-class correlation coefficients described. • Perceived Usefulness: The majority of both adolescents and staff reported finding the instrument useful and helpful.
Van der Helm et al., 2013 [35]	263	14 (2.5)	222 (84.4)	Secure residential youth care facility (61%) Youth prison (38%)	Residents of a secure youth facility and juvenile prison	Netherlands	ATMQ	• Face Validity: Not assessed. • Construct Validity: Confirmatory Factor Analysis (CFA) and Fit Indices showed a good fit to the data, indicating good construct validity. • Internal Consistency: Cronbachs alpha was good (α = 0.84) • Concurrent Validity: ATMQ items strongly correlated with related items on the Prison Group Climate Instrument suggesting good concurrent validity. • Discriminatory Validity: Treatment motivation items were not significantly associated with a measure of social desirability, which supports good discriminant validity. • Inter-rater Reliability: Not assessed. • Test–retest reliability: Not assessed. • Perceived Usefulness: Not assessed.
Van der Helm et al., 2013 [36]	128	15.7 (1.4)	79 (62)	Secure institutional youth care (82%) Youth Correctional Centers (18%).	Residents of these facilities	Netherlands	TOPS-A	• Face Validity: Not assessed. • Construct Validity: CFA showed that all questionnaire items and their corresponding factor loadings were significant, suggesting good construct validity. • Internal Consistency: Cronbach’s alpha for the overall scale of problematic social situations was (α = 0.90). • Concurrent Validity: Relevant TOPS-A items were significantly correlated with related measures of both aggression and living group climate suggesting moderate to strong concurrent validity. • Discriminatory Validity: Several relevant items were not significantly associated with a measure of social desirability, suggesting some discriminatory validity. • Inter-rater Reliability: Not assessed. • Test–retest reliability: Not assessed. • Perceived Usefulness: Not assessed.
van Nieuwenhuizen et al., 2013 [37]	133	17.3	100	Youth Forensic Psychiatric Hospital	Inpatients of this facility	Netherlands	FIOS	• Face Validity: Not assessed. • Construct Validity: CFA results indicated the goodness of fit indices for the original FIOS six-factor structure did not meet the required cut-off values, suggesting low construct validity However Exploratory Factor Analysis using a five-factor structure (removing insight items from the scale) had a good enough fit to the data (CFI = 0.93; TLI = 0.95 and RMSEA = 0.085). • Internal Consistency: Cronbach’s alpha was good for three areas on the scale (self-care [α = 0.84]; social behaviour [α = 0.85]; oppositional behaviour [α = 0.85]); was acceptable for one area (distress [ α = 0.77]; but was less than acceptable for the two remaining areas (insight [α = 0.84]; verbal skills [α = 0.84]). • Concurrent Validity: Items from only once area of the FIOS Scale (Distress) were significantly correlated with related items on a similar scale (internalising problems on the Youth Self Report (YSR) or Adult Self Report (ASR) scale). • Discriminatory Validity: Similarly, items from only one area of the FIOS (social behaviour) were significantly correlated with similar items on a related teacher-reported scale (i.e. externalising problems on the Teacher Report Form). • Inter-rater Reliability: Not assessed. • Test–retest reliability: Not assessed. • Perceived Usefulness: Not assessed.
Yates et al., 2006 [38]	64	14 (median)	67	Secure residential complex managed by social services	Residents of this complex who were either admitted through the social welfare system or were criminal admissions (on remand or sentenced).	England	HoNOSCA	• Face Validity: Not assessed. • Construct Validity: Not assessed. • Internal Consistency: Not assessed. • Concurrent Validity: Correlations between adolescent scores on the HoNOSCA and adolescent reported mood symptoms on the Moods and Feelings Questionnaire (MFQ) and on the emotional sub-scale of the Strengths and Difficulties Questionnaire were acceptable (*r* = 0.4, *P* < 0.0001). The authors therefore concluded that the HoNOSCA may be help in the identification of mental health problems amongst this population. However, staff reported HoNOSCA scores on admission showed poor and non-statistically significant with all areas of the SDQ and the MFQ. In addition, staff scores and adolescent scores were poorly correlated for all areas. Further validation studies to determine its concurrent and discriminatory value are therefore warranted. • Discriminatory Value: As above. • Inter-rater Reliability: Inter-rater reliability was good (α = 0.6–0.9, and *p* < 0.02 for all items). • Test–retest reliability: The HoNSCA is a dynamic measure and scores were shown to be sensitive to change. Follow-up HoNOSCA ratings (at 3 and 6 months) were reported to have been sensitive to change. • Perceived Usefulness: Not assessed.

Table 2 shows a summary of each included study that met our inclusion criteria. Included is details of sample size, mean age of population, sex, setting, participants, country of origin, outcome measurement tool used, and the conclusion of the study on validation for use of the outcome measure in an FCAMHS cohort

### Summary of included instruments

#### BARO

Doreleijers et al. performed a validation study of the BARO, a first-line screening instrument for the identification of psychiatric disorders, adverse environmental factors, and levels of (dys)function in adolescent offenders (age 12 to 18) [34]. The BARO was developed specifically for use with such young people, using risk factors well known from the literature and by means of secondary analyses of data from a psychiatric prevalence study in adjudicated adolescents [39]. In collaboration with the child protection board, a standard instrument layout was created by Doreleijers et al. and subsequently translated into English, German, Russian and Finnish. At the time of the included study, the BARO was being used in Switzerland, Austria and Finland [34]. The German-language version was validated in Switzerland, with results published in German language so not meeting our inclusion criteria [40]. This included study was carried out in order to assess the psychometric properties for psychiatric disorders and psychosocial problems, alongside the perceived usefulness of the BARO. Doreleijers and colleagues concluded that the BARO has sufficient to good psychometric properties including moderate to strong discriminatory value and is considered a good screening instrument by the Child Protection Board social workers [34]. With respect to reliability, it was demonstrated that information from both the youth and the parent is preferable to results from the youth only. Doreleijers postulated that this indicates that when parents are not available for screening purposes moderate discriminatory value for detecting psychiatric disorders and psychosocial problems can be obtained from the young person, but not the other way around. It was suggested that future research should focus on this area, because it may help to reduce the amount of information requested from each person, and subsequently the duration and personnel costs of the investigation [34].

#### ATMQ

Two identified studies looked at the ATMQ [32, 35], which was developed to investigate ‘readiness to change’, in relation to the final three stages of the model of Prochaska and DiClemente (preparation, action, and maintenance) [41]. Beyond ‘adolescent’ neither study suggests a specific age range that this tool was deemed suitable for, however the tools were validated on populations aged 12–20 [35] and 17–26 [32] respectively. Van de Helm et al. describe initial development of the ATMQ, for use in Dutch youth correctional facilities [35]. The ATMQ is an adapted version of the Motivation for Treatment Questionaire (MTQ), based on the Transtheoretical Motivation Model of Prochaska and DiClemente [41]. The MTQ had previously been validated for use with incarcerated adolescents, but the findings were published in Dutch and therefore did not meet the inclusion criteria of this review [42]. Van Der Helm and colleagues concluded that evidence for construct validity of the ATMQ and good internal consistency was found in a confirmatory factor analysis and reliability analyses [35].

Our second included study focusing on the ATMQ was conducted by Heynen et al., and described testing of the construct validity of the translated German version of the ATMQ by means of a confirmatory factor analysis. This establishes concurrent validity by examining the relation between treatment motivation and living group climate (designated by growth, positive support, a good atmosphere and low repression) in incarcerated German juvenile offenders. Heynen concluded that the ATMQ can be used to validly and reliably assess treatment motivation within juvenile justice facilities in Germany [32]. Heynen postulates that the German version of the ATMQ can be used as a basis to target rehabilitation of delinquent youth. This short measurement instrument to investigate treatment motivation is beneficial as it allows for simple, repeated measurements over a short period of time. This was seen to be especially important in this specific FCAMHS setting where the stay of young people in detention mostly covered a short period of time (90 days–9 month). The ATMQ could be an important instrument not only to investigate treatment effects in youth prison, but also to assess the therapeutic effects of group treatment in terms of treatment motivation itself [32].

#### HoNOSCA

Two included studies assessed the HoNOSCA, which measures behaviour, impairment, symptoms and social functioning from the practitioners’ perspective [20, 38]. The HoNOSCA is an adapted version of the HoNOS, specifically targeted at young people. In a general CAMHS population, the practitioner and parent tool can be used in relation to children aged 5 to 18 years [17]. The two included studies which took place in FCAMHS populations, looked at populations aged 13 to 18 [20] and 10 to 17 [38] respectively. Hunt and colleagues concluded that the inter-rater reliability of the HoNOSCA total scores and domain scores has been demonstrated to be reasonably high, with concurrent validity in relation to other relevant outcome measures, however several issues were highlighted with regard to assessing outcome in this setting [20]. One example of an issue encountered was a ‘ceiling effect’ where clients have a tendency to display more extreme behaviours and even if such behaviours are to significantly reduce in frequency or severity they will still score the maximum number on the scale. Another issue when applying the HoNOSCA to an FCAMHS population was the concept ‘drift’, where raters tend to underscore items due to a degree of desensitisation to severe clients over prolonged periods. Severely ill patients can therefore be scored at a lower level than is indicated by the scoring criteria. It was also deemed to be more challenging to score factors such as substance misuse (strict security measures almost totally eliminated this), family life and relationships (as most clients had experienced a disintegrated family background) and school attendance (as most clients did not attend school due to their incarceration). Hunt et al. made suggestions around modifications to the questionnaire, to make the scale more suitable to this patient group, for example instead looking at attachment of clients to their current carer (i.e. healthcare staff) or looking at attendance and participation in their treatment programme [20].

Yates and colleagues concluded that the HoNOSCA can be used reliably and is sensitive to change in a social services secure unit, however, correlation with adolescent-completed psychological measures was poor [38]. It was concluded that included adolescents might have been not adept at completing questionnaires, as many had learning difficulties. It was suggested that further research to examine the reasons for and implications for differences in views by staff and adolescents was called for [38]. Yates et al. did not comment on potential areas for improving specific scoring elements of the HoNOSCA.

#### S.NASA

Kroll and colleagues examined the S.NASA, which covers 21 areas of functioning including social, psychiatric, educational and life skills [33]. This instrument was developed specifically by Kroll’s research team, and subsequently validated in this included study. The S.NASA incorporated and adapted three well established adult needs assessment instruments, to assess areas of functioning including social, psychiatric, educational and life skills in adolescents. Beyond the use of the term ‘adolescents’ no specific suitable age group is suggested for this tool, however the study was carried out on young people aged betwwen 12.3 and 17.3 years old. Kroll concluding that it had moderate to good inter-rater and test-retest reliability coefficients, and good consensual and face validity [33]. It was intended that the schedule can be used by both health and non-health professionals, and is brief enough to be used in service based research and possibly some very specialized centres with an established research infrastructure. However, a limitation discussed is that the length and formal structure of the interview required makes it unlikely that many clinical services will use it routinely. However, a flexible clinical version is mentioned by Kroll [33].

#### TOPS-A

The development of TOPS-A as a new outcome measure is described specifically in this included study by Van der Helm et al., as an adaptation of the Taxonomy of Problematic Social Situations, which was developed for use in elementary school children in a standard, non- forensic population [36]. The TOPS-A is an observation instrument used to assess situation-specific social skill deficits in delinquent adolescents, specifically for use in secure institutional youth care. At the time of the study, the TOPS-A was already being used to assess problem levels and social skills training outcomes of adolescents who are involved in the criminal justice system and have a mild intellectual disability. The TOPS-A purports to measure inappropriate responses of adolescents (aged 14 to 18 years old) in four types of problematic social situations: situations of disadvantage, competition, accepting/giving help, and accepting authority. Van der Helm et al. and colleagues found evidence for construct validity and good internal consistency. Divergent validity could not be reliably assessed [36]. It is suggested that the TOPS-A can be used to target specific problem situations and inappropriate reactions, often encountered outside residential institutions [36]. Perhaps this could include other FCAMHS settings, however further validation would be required in any potential identified cohort.

#### FIOS

Nieuwenhuizen et al. performed a validation study of the FIOS, which was developed to assess the level of functioning of forensic psychiatric patients and is divided in six subscales: self-care (7 items), social behaviour (6 items), oppositional behaviour (10 items), insight offense/problems (4 items), verbal skills (3 items) and distress (5 items) [37]. The FIOS does not focus on psychiatric symptoms per se, but on general behaviour which is considered relevant to leading a life without being a threat to self and/or others [37]. Prior to Nieuwenhuizen’s 2011 study, it has only been used among adult forensic psychiatric patients and this was the first study in which the FIOS was used with young people. The study concluded that the value of the FIOS lies in the focus on behavioral functioning of ‘youngsters’ with judicial measures, with the setting for the study being a young forensic psychiatry hospital caring for males aged between the age of 16 to 24 years.

The results of this study suggested that the FIOS can be used in a population of young people and that it has, with some slight adjustments, good internal consistency and a stable factor structure. Nieuwenhizen concluded that 26 items, instead of the 35 items of the original version of the FIOS, seemed sufficient enough to score the behaviour of youngsters. Nine items (including all of the insight scale) were excluded as confirmatory factor analysis concluded that the goodness of fit indices for the original FIOS six-factor structure did not meet the required cut-off values. The study did not suggested reasons why these nine items may not have been relevant to the FCAMHS population. However, it was suggested that reducing the number of items would allow for customization of the instrument more for an adolescent population. For instance, by adding items dealing with family and peer influence and drug use [37].

### Quality appraisal

Articles scored between 17/40 and 30/40 on the CCAT (see Table 3). One included study was rated as LQ [38], six included studies were rated as MQ [20, 32–36], and one study was rated as HQ [37]. No studies were rated as VHQ. The study rated as LQ posed an unclear background for proposing the research question and had limited in depth information on the design of the study. Results and discussion were brief with a lack of in-depth analysis. It should be noted however that this has been published as a ‘short report’ meaning that there was likely a more limited word count in the journal’s submission requirements and so less data could be shared. The studies rated as MQ contained methodological issues such as not reporting in detail how the samples were recruited. Results and discussion were explained in more depth however there were several areas not clearly covered such as sources of bias. The study rated as HQ included most detail about the research methodology and interpretation of results, but there was some missing information on sampling, data collection and ethical matters.

Potential sources of authorship bias/ conflict of interest, are summarised in Table 4. In six included studies, the research team carrying out the study were also involved in creating the measuring instrument being validated [32–37]. Two studies didt not declare any information on funding [20, 32]. Four studies were funded by national research foundations [34–36]. The final two studies were funded by relevant national health service boards [33, 38].

**Table 3:** Quality appraisal of included studies using CCAT

Reference	Item descriptor								Total score	Quality of evidence
	Preliminaries	Introduction	Design	Sampling	Data collection	Ethical matters	Results	Discussion		
Doreleijers et al., 2011 [34]	4/5	5 /5	4/5	2/5	2/5	1/5	2/5	2/5	22/40	Moderate
Heynen et al., 2017 [32]	4/5	5/5	4/5	3/5	2/5	3/5	3/5	3/5	27/40	Moderate
Hunt et al., 2009 [20]	4/5	5/5	3/5	3/5	4/5	4/5	3/5	3/5	29/40	Moderate
Kroll et al., 1999 [33]	4/5	3/5	3/5	3/5	3/5	3/5	2/5	2/5	23/40	Moderate
Van der Helm et al., 2013 [35]	4/5	5/5	3/5	3/5	3/5	4/5	3/5	4/5	29/40	Moderate
Van der Helm et al., 2013 [36]	3/5	4/5	3/5	3/5	3/5	4/5	3/5	4/5	27/40	Moderate
van Nieuwenhuizen et al., 2013 [37]	4/5	5/5	4/5	3/5	3/5	3/5	4/5	4/5	30/40	High
Yates et al., 2006 [38]	2/5	4/5	2/5	2/5	2/5	1/5	2/5	2/5	17/40	Low

Table 3 shows the CCAT score for each included study, across each individual item descriptor on the CCAT, as well as the total score. The quality of evidence is described based on a rating of low/ moderare/ high or very high quality

**Table 4:** Potential authorship bias

Reference	Potential Authorship Bias (Conflict of interest)	
	Non- financial	Financial
Doreleijers et al., 2011 [34]	The authors declare that they have no competing interests, however team carrying out study were also involved in creating measuring instrument being validated.	The article processing charge of this manuscript has been funded by the German Research Foundation.
Heynen et al., 2017 [32]	Team carrying out study were also involved in translating measuring instrument being validated.	No information on funding.
Hunt et al., 2009 [20]	Not overtly stated but nil evident.	No information on funding.
Kroll et al., 1999 [33]	Team carrying out study were also involved in translating measuring instrument being validated.	This study was funded by Salford Mental Health Trust.
Van der Helm et al., 2013 [35]	The authors declared no potential conflicts of interest with respect to the research, authorship, and/or publication of this article, however team carrying out study were also involved in creating measuring instrument being validated.	This article was partly financed by a ‘Raak Publiek’ grant.
Van der Helm et al., 2013 [36]	The authors declared no potential conflicts of interest with respect to the research, authorship, and/or publication of this article, however team carrying out study were also involved in creating measuring instrument being validated.	This article was partly financed by a ‘Sia-Raak Publiek’ grant.
van Nieuwenhuizen et al., 2013 [37]	The authors declare that they have no competing interests however noted modification made by authors to modify outcome measure to use 26 items, instead of the 35 items of the original version of the FIOS.	The article processing charge of this manuscript has been funded by the German Research Foundation.
Yates et al., 2006 [38]	Not overtly stated but nil evident.	The study was funded by a grant from High Security Psychiatric Services Commissioning Board Research and Development.

Table 4 shows potential sources of authorship bias, in the form of potential non-financial and financial conflicts of interest mentioned in each paper

## Discussion

This systematic review aimed to provide an overview of instruments which have been validated for use as outcome measures in child and adolescent forensic mental health services. There is limited evidence of specific validation of structured instruments as outcome measures within an FCAMHS population, and outcome measures from included studies focus on the areas of treatment motivation, level of functioning, psychiatric symptoms, care needs and responses to social situations.

When considering the wide range of outcome and experience measures that have been verified as validated by CORC [17], only the HoNOSCA has been validated within an FCAMHS population. Other commonly used outcome assessment and experience measures including the Childrens Global Assessment Scale (CGAS), Experience of Service Questionnaire (ESQ), Revised Children’s Anxiety and Depression Scale (and Subscales) (RCADS) and the Strength and Difficulties Questionnaire (SDQ) do not appear to have been specifically validated in an FCAMHS population. A summary of the instruments mentioned in this paper is included in Table 5.

**Table 5:** Summary on validation of instruments mentioned in paper

Instrument	Validated for use in FCAMHS population	Mentioned as validated by CORC for generic CAMHS population
BARO	YES	NO
ATMQ	YES	NO
HoNOSCA	YES	YES
S.NASA	YES	NO
TOPS-A	YES	NO
FIOS	YES	NO
CGAS	NO	YES
ESQ	NO	YES
RCADS	NO	YES
SDQ	NO	YES
GBO	NO	YES

Table 5 shows each instrument which has been mentioned in this paper, alongside whether the instrument has been validated for use in the FCAMHS population, and/ or mentioned as validated by CORC for use in the generic CAMHS population

Our broad definition of what constitutes a structured instrument ensured a wide range of instruments were considered. Included papers were analysed using a recognised quality assessment process. To our knowledge, this is the first time that such a systematic approach has been applied to the analysis of research on outcome measures in FCAMHS [25].

### Implications for research

For the studies that met the criteria for inclusion in this systematic review, the sample size is generally low (ranging from 24 to 295). In these studies, only specific psychometric properties of the included outcome measures have been methodologically validated, and thus further studies are required specifically focusing on other areas of validation. Much of the evidence identified was produced by the teams that originally developed the instruments. Sufficient validation studies should therefore be conducted independently of the original authors.

We note Ryland’s 2021 systematic review [15] on outcome measures in adult forensic mental health services. We would draw similar conclusions in relation to implications of our findings to future research. The lack of validation studies involving structured outcome assessment tools which are currently more commonly used among the FCAMHs population was unexpected, particularly given the age and popularity of these instruments. For example, no validation studies of the SDQ, Experience of Service Questionnaire (ESQ), CGAS or Goal Based Outcomes (GBO) were identified during this review. However, this may reflect the diversity of their intended uses [15]. Testing of comprehensiveness and relevance should be completed in the population for which outcome measurement instruments are intended [43]. This can take place after the instrument is available in its final form and does not have to occur contemporaneously with development [44]. Further research is therefore necessary to establish the content validity of instruments which are to be used as outcome measures in FCAMHS.

Whilst there are a multitude of current outcome assessment tools available, without adequate validation studies it cannot be certain that such tools are valid for use in an FCAMHS population. An alternative approach would be to develop new instruments for this population, with a specific focus on the unique presentation and needs of such a population. Such tools should be developed according to the latest best practice guidelines and involve co-production with relevant stakeholders including young people and clinicians, to identify instuments that fit the needs of both the individual and the service [18, 43].

### Implications for policy and practice

Our findings are of direct clinical relevance, as results will help guide future clinician choice when selecting structured instruments as outcome measures for use within FCAMHS. This review identified eight instruments that have been validated for use as outcome measures in the FCAMHS setting. The use of instruments can differ considerably between clinician and setting. Services should therefore start by deciding which outcomes are important in their population, before selecting high quality outcome measures that cover such outcomes in a way that is practical to implement [15]. It can be challenging for a service to decide which outcome measures to focus on however, when so few have been validated in the relevant population.

It also must be acknowledged that the studies which met our inclusion criteria took place across a variety of settings including youth prison, inpatient psychiatric secure units, secure residential youth care and outpatients. There are significant differences in a young persons needs (both mental health and criminogenic) between such settings, and effort should be made to further development in developing pathways and understanding such needs [4, 13]. In considering the difference in mean ages between included studies (ranging from 14 to 20.3 years), we must acknowledge the variation in developmental and clinical needs in our included population. This could be considered a strength of this study, as a wider age range has been captured, so findings will likely be more applicable to different clinical populations. Young people can move between such settings, and having validated outcome measures that could assess their longitudinal progress would be of value [13]. Further research studies would help clinicians involved in such services decide on what tools should be used, based on the large number available [17].

### Limitations

In our search strategy, we utilised COSMIN listed translations of the PubMed search filter for finding studies on measurement properties of measurement instruments [30]. These translations were generally not validated, and therefore may have missed some relevant studies. We have excluded studies which focused solely on risk, personality traits and intelligence which are still very relevant to service user outcomes however tend to be less dynamic. We have also excluded studies on group rather than individual progress or outcomes which could miss some important collective outcome data. We excluded non-peer-reviewed literature so there is a possibility that other validation studies have been described in relevant product literature. Limiting the search to papers where the full text is available in English may have limited findings from some areas across the world, meaning results may be less applicable to such settings. All included studies took place in Western Europe.

Heynen and colleagues included data from a proportion of participants who were aged over 18 [32]. Kroll and colleagues included some participants aged under 18 who were cared for in general adolescent psychiatric services [33]. Therefore, results from both these studies may not necessarily be fully generalisable to an FCAMHS specific cohort. Structured instruments as outcome measures have been more frequently validated within the general CAMHS cohort [17]. In relation to our quality appraisal of included studies using the CCAT, it is acknowledged that inter-rater reliability was high but could not be formally calculated.

## Conclusions

There are many instruments available that can be used as outcome measures in child and adolescent mental health services, however only a small number of these instruments have been validated specifically for use with an FCAMHS population. Even for instruments that have been validated in this population, evidence is limited and specific to a small number of mostly low to moderate quality studies. Additional research is required to validate whether current outcome assessment tools are applicable to the FCAMHS population. If current tools are deemed to not be valid for use in this population, an alternative would be the development of new FCAMHS specific outcome measurement tools. Whatever approach is taken, input from relevant stakeholder groups, especially young people and their carers should be encouraged. This should follow current best practice guidelines for outcome measure development [2, 43].

## Electronic Supplementary Material

Below is the link to the electronic supplementary material.

Supplementary Material 1

## Data Availability

No datasets were generated or analysed during the current study.
