# Supplementary material for: Identifying studies examining the validity of instruments for use as outcome measures in child and adolescent forensic mental health services: a systematic review
Source: Eur Child Adolesc Psychiatry. 2024 Aug 7;34(2):519–33. doi: 10.1007/s00787-024-02514-7 (PMC11868235; doi:10.1007/s00787-024-02514-7)
Supplement: Supplementary file 1 — Supplementary Material 1 [file 787_2024_2514_MOESM1_ESM.docx]

**Appendix 1: Full Search Strategy**

**OVID Embase**

**Search 1: Child and adolescent cohort:**

exp child/

OR exp adolescent/

OR (child* or adolesc* or teenage* or juvenile or (young adj1 person*) or (young adj1 people) or youth*).mp. [mp=title, abstract, heading word, drug trade name, original title, device manufacturer, drug manufacturer, device trade name, keyword heading word, floating subheading word, candidate term word]

**Search 2: Forensic/ secure cohort:**

exp "Forensic Psychiatry"/

OR ((secure OR forensic ) ADJ3 (unit OR ward OR hospital OR department OR service* OR psych* OR team )).mp.

**Search 3: Mental illness or Neurodevelopmental disorder:**

exp mental disease/

OR exp adolescent depression/ or exp depression/ or exp atypical depression/

OR exp anxiety/ or exp anxiety disorder/ or exp generalized anxiety disorder/

OR exp posttraumatic stress disorder/

OR exp schizophrenia/ or exp schizoaffective psychosis/ or exp psychosis/ or exp obsessive compulsive disorder/

OR exp memory/

OR exp apraxia/

OR exp language disability/

OR exp Intellectual Disability/

OR exp Child Development/

OR exp Autistic Disorder/

OR exp Attention Deficit Disorder with Hyperactivity/

OR exp Apraxias/

OR exp Memory/

OR exp Language Disorders/

OR exp Executive Function/

OR exp Developmental Disorder

OR exp fetal alcohol syndrome/

OR ((affective or emotional* or mental* or psychological*) adj2 (disease? or disorder? or health* or ill* or well* or stress* or distress* or trauma*)).mp.

OR (acute stress or adjustment disorder* or ADNOS or affective disorder* or agoraphobi* or phobi* or anxiety disorder? or GAD or (combat adj (disorder* or fatigue or neuros* or syndrome*)) or compulsi* or (obsessive adj2 disorder*) or OCD or depressed or depression or depressive? or dysphori* or dysthymi* or melanchol* or emotional trauma or fear or health anxiety or hysteri* or MDD or mental* or mood? or neurastheni* or neurotic or neuros* or panic or ((post-trauma* or posttrauma* or post trauma) adj stress*) or flashback* or (trauma* adj (nightmare* or stress or neuros*)) or (psych* adj (stress or trauma*)) or psychotrauma* or ((sever* or serious* or major* or chronic* or complex* or critical* or endur* or persist* or resist* or acute) adj2 (anxiety or psycholog*)) or psychiatr* or psychopathol* or PTSD).mp.

OR "Schizophrenia Spectrum and Other Psychotic Disorder*".mp.

OR psychotic disorder*.mp.

or schizophreni*.mp.

or schizoaffective.mp.

or schizophreniform.mp.

or reactive psychosis.mp.

OR psychoses.mp.

OR reactive psychoses.mp.

OR (intellectual$ adj impair$).mp.

OR IQ.mp.

OR (intellectual adj ability).mp.

OR neurodevelopment*.mp.

OR (mental$ adj retard$).mp.

OR child development.mp.

OR (autism or autistic).mp.

OR attention deficit.mp.

OR dyspraxia.mp.

OR memory.mp.

OR executive function$.mp.

OR (fetal alcohol syndrome OR foetal alcohol syndrome OR fetal alcohol OR foetal alcohol OR fasd OR fae OR arbd OR arnd OR prenatal alcohol exposure OR pae OR alcohol exposed pregnancy OR aep).mp

**Search 4: COSMIN filter for measuring properties of measuring instruments:**

(instrumentation or methods).sh.

OR exp Psychometrics/

OR psychometr*.tw.

OR (clinimetr* or clinometr*).mp.

OR exp Outcome Assessment, Health Care/

OR outcome assessment.tw.

OR outcome measure*.mp.

OR exp Observer Variation/

OR observer variation.tw.

OR exp Health Status Indicators/

OR exp Reproducibility of Results/

OR reproducib*.tw.

OR exp Discriminant Analysis/

OR (reliab* or unreliab* or valid* or coefficient of variation or coefficient or homogeneity or homogeneous or internal consistency).tw.

OR (cronbach* and (alpha or alphas)).tw.

OR item and (correlation* or selection* or reduction*)).tw.

OR agreement.mp.

OR precision.mp.

OR imprecision.mp.

OR precise values.mp.

OR test-retest.tw.

OR (test and retest).tw.

OR (reliab* and (test or retest)).tw.

OR stability.tw.

OR (interrater or inter-rater or intrarater or intra-rater).tw.

OR (intertester or inter-tester or intratester or intra-tester).tw.

OR (interobserver or inter-observer or intraobserver or intra-observer).tw.

OR (intertechnician or inter-technician or intratechnician or intra-technician).tw.

OR (interexaminer or inter-examiner or intraexaminer or intra-examiner).tw.

OR (interassay or inter-assay or intraassay or intra-assay).tw.

OR (interindividual or inter-individual or intraindividual or intra-individual).tw.

OR (interparticipant or inter-participant or intraparticipant or intra-participant).tw.

OR kappa.tw.

OR kappas.tw.

OR repeatab*.mp.

OR ((replicab* or repeated) and (measure or measures or findings or result or results or test or OR tests)).mp.

OR (generaliza* or generalisa*).tw.

OR concordance.tw.

OR (intraclass and correlation*).tw.

OR discriminative.tw.

OR known group.tw.

OR (factor analysis or factor analyses or factor structure or factor structures).tw.

OR dimension*.tw.

OR subscale*.tw.

OR (multitrait and scaling and (analysis or analyses)).tw.

OR item discriminant.tw.

OR interscale correlation*.tw.

OR (error or errors).tw.

OR individual variability.tw.

OR interval variability.tw.

OR rate variability.tw.

OR (variability and (analysis or values)).tw.

OR (uncertainty and (measurement or measuring)).tw.

OR standard error of measurement.tw.

OR sensitiv*.tw.

OR responsive*.tw.

OR (limit and detection).tw.

OR minimal detectable concentration.tw.

OR interpretab*.tw.

OR ((minimal or minimally or clinical or clinically) and (important or significant or

OR detectable) and (change or difference)).tw.

OR (small* and (real or detectable) and (change or difference)).tw.

OR meaningful change.tw.

OR ceiling effect.tw.

OR floor effect.tw.

OR item response model.tw.

OR IRT.tw.

OR rasch.tw.

OR differential item functioning.tw.

OR DIF.tw.

OR computer adaptive testing.tw.

OR item bank.tw.

OR cross-cultural equivalence.tw.

**Search 1: Child and adolescent cohort AND Search 2: Forensic/ secure cohort AND Search 3: Mental illness or Neurodevelopmental disorder AND Search 4: COSMIN filter for measuring properties of measuring instruments** NOT (address or biography or "case reports" or comment or directory or editorial or festschrift or interview or lecture or "legal case" or legislation or letter or news or "newspaper article" or "patient education handout" or "popular work" or congress or "consensus development conference" or "consensus development conference, NIH" or "practice guideline").mp.

**OVID Medline**

**Search 1: Child and adolescent cohort:**

exp child/

OR exp adolescent/

OR ((child* or adolesc* or teenage* or juvenile or (young adj1 person*) or (young adj1 people) or youth*).mp

**Search 2: Forensic/ secure cohort:**

exp "Forensic Psychiatry"/

OR ((secure OR forensic ) ADJ3 (unit OR ward OR hospital OR department OR service* OR psych* OR team )).mp.

**Search 3: Mental illness or Neurodevelopmental disorder:**

exp mental disease/ OR exp depression/ or exp atypical depression/ or exp anxiety/ or exp anxiety disorder/ or exp generalized anxiety disorder/ or exp posttraumatic stress disorder/

OR "mental disease"/ OR "mental disorders"/ OR "mental health"/

anxiety/ OR "anxiety disorder"/ OR depression/ OR "agitated depression"/ OR "atypical depression"/ OR "chronic depression"/ OR dysphoria/ OR dysthymia/ OR "endogenous depression"/ OR "involutional depression"/ OR "major depression"/ OR melancholia/ OR "minor depression"/ OR "organic depression"/ OR "postoperative depression"/ OR "reactive depression"/ OR "recurrent brief depression"/ OR "subsyndromal depression"/ OR "treatment resistant depression"/ OR neurosis/ OR "affective neurosis"/ OR "anxiety neurosis"/ OR hysteria/ OR neurasthenia/ OR psychasthenia/ OR "adjustment disorder"/ OR "anxiety disorder"/ OR "acute stress disorder"/ OR "distress syndrome"/ OR "generalized anxiety disorder"/ OR panic/ OR "posttraumatic stress disorder"/ OR "separation anxiety"/ OR "obsessive compulsive disorder"/ OR compulsion/ OR obsession/ OR phobia/ OR agoraphobia/ OR claustrophobia/ OR neophobia/ OR "social phobia"/ OR "masked depression"/ OR "mood disorder"/ OR "major affective disorder"/ OR "minor affective disorder"/ OR "emotional disorder"/

OR "mood disorders"/ OR depression/ OR "depressive disorder"/ OR "depressive disorder, major"/ OR "depressive disorder, treatment-resistant"/ OR "dysthymic disorder"/ OR "anxiety disorders"/ OR agoraphobia/ OR "anxiety, separation"/ OR "neurocirculatory asthenia"/ OR "neurotic disorders"/ OR "obsessive-compulsive disorder"/ OR "hoarding disorder"/ OR "panic disorder"/ OR "phobic disorders"/ OR "phobia, social"/ OR "trauma and stressor related disorders"/ OR "adjustment disorders"/ OR "stress disorders, traumatic"/ OR "combat disorders"/ OR "psychological trauma"/ OR "stress disorders, post-traumatic"/ OR "stress disorders, traumatic, acute"/

OR exp Autism Spectrum Disorder/ or exp Neurodevelopmental Disorders/ or exp Developmental Disabilities/

OR exp "Attention Deficit Disorder with Hyperactivity"/

OR exp "Intellectual Disability"/

OR exp "Autistic Disorder"/

OR exp "Child Development"/

OR exp "Language Disorders"/

OR exp "Executive Function"/

OR exp Fetal Alcohol Spectrum Disorders/

OR exp ACUTE PSYCHOSIS/ or exp CHRONIC PSYCHOSIS/ or exp PSYCHOSIS/ or psychosis.mp.

OR exp Schizophrenia/

OR exp SCHIZOAFFECTIVE DISORDER/

OR exp SCHIZOPHRENIFORM DISORDER/

OR exp Reactive Psychosis/

OR ((affective OR emotional* OR mental* OR psychological* ) ADJ2 (disease? OR disorder? OR health* OR ill* OR well* OR stress* OR distress* OR trauma* )).mp.

OR ("acute stress" OR "adjustment disorder*" OR ADNOS OR "affective disorder*" OR agoraphobi* OR phobi* OR "anxiety disorder?" OR GAD OR (combat ADJ (disorder* OR fatigue OR neuros* OR syndrome* )) OR compulsi* OR (obsessive ADJ2 disorder* ) OR OCD OR depressed OR depression OR depressive? OR dysphori* OR dysthymi* OR melanchol* OR "emotional trauma" OR fear OR "health anxiety" OR hysteri* OR MDD OR mental* OR mood? OR neurastheni* OR neurotic OR neuros* OR panic OR ((post-trauma* OR posttrauma* OR "post trauma" ) ADJ stress* ) OR flashback* OR (trauma* ADJ (nightmare* OR stress OR neuros* )) OR (psych* ADJ (stress OR trauma* )) OR psychotrauma* OR ((sever* OR serious* OR major* OR chronic* OR complex* OR critical* OR endur* OR persist* OR resist* OR acute ) ADJ2 (anxiety OR psycholog* )) OR psychiatr* OR psychopathol* OR PTSD ).mp.

OR "Schizophrenia Spectrum and Other Psychotic Disorder*".mp.

OR psychotic disorder*.mp.

or schizophreni*.mp.

or schizoaffective.mp.

or schizophreniform.mp.

or reactive psychosis.mp.

OR psychoses.mp.

OR reactive psychoses.mp.

OR (intellectual$ ADJ impair$).mp

OR IQ.mp

OR (intellectual ADJ ability ).mp

OR neurodevelopment.mp

OR (mental$ ADJ retard$ ).mp

OR "child development".mp

OR (autism OR autistic ).mp

OR "attention deficit".mp

OR dyspraxia.mp

OR memory.mp

OR "executive function$".mp

OR (fetal alcohol syndrome or foetal alcohol syndrome or fetal alcohol or foetal alcohol or fasd or fae or arbd or arnd or prenatal alcohol exposure or pae or alcohol exposed pregnancy or aep).mp.

**Search 4: COSMIN filter for measuring properties of measuring instruments:**

(instrumentation or methods).sh.

OR (validation study or comparative study).pt.

OR exp Psychometrics/

OR psychometr*.tw.

OR (clinimetr* or clinometr*).mp.

OR exp Outcome Assessment, Health Care/

OR outcome assessment.tw.

OR outcome measure*.mp.

OR exp Observer Variation/

OR observer variation.tw.

OR exp Health Status Indicators/

OR exp Reproducibility of Results/

OR reproducib*.tw.

OR exp Discriminant Analysis/

OR (reliab* or unreliab* or valid* or coefficient of variation or coefficient or homogeneity or homogeneous or internal consistency).tw.

OR (cronbach* and (alpha or alphas)).tw.

OR (item and (correlation* or selection* or reduction*)).tw.

OR agreement.mp.

OR precision.mp.

OR imprecision.mp.

OR precise values.mp.

OR test-retest.tw.

OR (test and retest).tw.

OR (reliab* and (test or retest)).tw.

OR stability.tw.

OR (interrater or inter-rater or intrarater or intra-rater).tw.

OR (intertester or inter-tester or intratester or intra-tester).tw.

OR (interobserver or inter-observer or intraobserver or intra-observer).tw.

OR (intertechnician or inter-technician or intratechnician or intra-technician).tw.

OR (interexaminer or inter-examiner or intraexaminer or intra-examiner).tw.

OR (interassay or inter-assay or intraassay or intra-assay).tw.

OR (interindividual or inter-individual or intraindividual or intra-individual).tw.

OR (interparticipant or inter-participant or intraparticipant or intra-participant).tw.

OR kappa.tw.

OR kappas.tw.

OR repeatab*.mp.

OR ((replicab* or repeated) and (measure or measures or findings or result or results or test or tests)).mp.

OR (generaliza* or generalisa*).tw.

OR concordance.tw.

OR (intraclass and correlation*).tw.

OR discriminative.tw.

OR known group.tw.

OR (factor analysis or factor analyses or factor structure or factor structures).tw.

OR dimension*.tw.

OR subscale*.tw.

OR (multitrait and scaling and (analysis or analyses)).tw.

OR item discriminant.tw.

OR interscale correlation*.tw.

OR (error or errors).tw.

OR individual variability.tw.

OR interval variability.tw.

OR rate variability.tw.

OR (variability and (analysis or values)).tw.

OR (uncertainty and (measurement or measuring)).tw.

OR standard error of measurement.tw.

OR sensitiv*.tw.

OR responsive*.tw.

OR (limit and detection).tw.

OR minimal detectable concentration.tw.

OR interpretab*.tw.

OR ((minimal or minimally or clinical or clinically) and (important or significant or detectable) and (change or difference)).tw.

OR (small* and (real or detectable) and (change or difference)).tw.

OR meaningful change.tw.

OR ceiling effect.tw.

OR floor effect.tw.

OR item response model.tw.

OR IRT.tw.

OR rasch.tw.

OR differential item functioning.tw.

OR DIF.tw.

OR computer adaptive testing.tw.

OR item bank.tw.

OR cross-cultural equivalence.tw.

**Search 1: Child and adolescent cohort AND Search 2: Forensic/ secure cohort AND Search 3: Mental illness or Neurodevelopmental disorder AND Search 4: COSMIN filter for measuring properties of measuring instruments** NOT (address or biography or case reports or comment or directory or editorial or festschrift or interview or lecture or legal case or legislation or letter or news or newspaper article or patient education handout or popular work or congress or consensus development conference or consensus development conference, NIH or practice guideline).pt.

**PsycINFO**

**Search 1: Child and adolescent cohort:**

exp Child Psychiatry/ or exp Child Psychopathology/

exp Adolescent Psychiatry/ or exp Adolescent Psychopathology/

OR (child* or adolesc* or teenage* or juvenile or (young adj1 person*) or (young adj1 people) or youth*).mp

**Search 2: Forensic/ secure cohort:**

exp "Forensic Psychiatry"/

OR ((secure or forensic) adj3 (unit or ward or hospital or department or service* or psych* or team)).mp.

**Search 3: Mental illness or Neurodevelopmental disorder:**

exp Mental Disorders or exp Schizophrenia/ or exp Major Depression/ or exp Mental Health/

or exp Atypical Depression/ or exp Anxiety Disorders/ or exp Posttraumatic Stress Disorder/ or exp Phobias/ or exp Compulsions/ or exp Obsessions/ or exp Hoarding Behavior/ or exp Autism Spectrum Disorders/ or exp Intellectual Development Disorder/ or exp Developmental Disabilities/ or exp Attention Deficit Disorder with Hyperactivity/or exp Language Disorders/or exp Bipolar Disorder/ or exp Obsessive Compulsive Disorder/ or exp Schizophrenia/ or exp Psychosis/ or exp Fetal Alcohol Syndrome/

OR ("acute stress" or "adjustment disorder*" or ADNOS or "affective disorder*" or agoraphobi* or phobi* or "anxiety disorder?" or GAD or (combat adj (disorder* or fatigue or neuros* or syndrome*)) or compulsi* or (obsessive adj2 disorder*) or OCD or depressed or depression or depressive? or dysphori* or dysthymi* or melanchol* or "emotional trauma" or fear or "health anxiety" or hysteri* or MDD or mental* or mood? or neurastheni* or neurotic or neuros* or panic or ((post-trauma* or posttrauma* or "post trauma") adj stress*) or flashback* or (trauma* adj (nightmare* or stress or neuros*)) or (psych* adj (stress or trauma*)) or psychotrauma* or ((sever* or serious* or major* or chronic* or complex* or critical* or endur* or persist* or resist* or acute) adj2 (anxiety or psycholog*)) or psychiatr* or psychopathol* or PTSD).mp.

OR ("Schizophrenia Spectrum and Other Psychotic Disorder*" or psychotic disorder* or schizophreni* or schizoaffective or schizophreniform or reactive psychosis or psychoses or reactive psychoses).mp

OR (psychiatr* or psychopathol* or PTSD or "Intellectual Disability" or (intellectual? adj1 impair?) or IQ or (intellectual adj1 ability) or neurodevelopment or "Child Development").mp.

OR ("Autistic Disorder" or (autism or autistic) or "Attention Deficit Disorder with Hyperactivity" or "attention deficit" or Apraxias or dyspraxia or "Language Disorders" or "Executive Function" or "executive function").mp.

OR (fetal alcohol syndrome OR foetal alcohol syndrome OR fetal alcohol OR foetal alcohol OR fasd OR fae OR arbd OR arnd OR prenatal alcohol exposure OR pae OR alcohol exposed pregnancy OR aep).mp

**Search 4: COSMIN filter for measuring properties of measuring instruments:**

Interrater Reliability/

OR exp Psychometrics/

OR exp Measurement/ or exp Test Validity/

OR exp Treatment Effectiveness Evaluation/ or exp Treatment Outcomes/

OR exp Test Validity/ or exp Test Reliability/

OR (instrumentation or methods or (validation study or comparative study) or (clinimetr* or clinometr*) or outcome measure* or agreement or precision or imprecision or precise values or repeatab* or ((replicab* or repeated) and (measure or measures or findings or result or results or test or tests))).mp.

OR (outcome assessment or observer variation or reproducib* or (reliab* or unreliab* or valid* or coefficient of variation or coefficient or homogeneity or homogeneous or internal consistency) or (cronbach* and (alpha or alphas)) or (item and (correlation* or selection* or reduction*)) or test-retest or (test and retest) or (reliab* and (test or retest)) or stability or (interrater or inter-rater or intrarater or intra-rater) or (intertester or inter-tester or intratester or intra-tester) or (interobserver or inter-observer or intraobserver or intra-observer) or (intertechnician or inter-technician or intratechnician or intra-technician) or (interexaminer or inter-examiner or intraexaminer or intra-examiner) or (interassay or inter-assay or intraassay or intra-assay) or (interindividual or inter-individual or intraindividual or intra-individual) or (interparticipant or inter-participant or intraparticipant or intra-participant) or kappa or kappas or (generaliza* or generalisa*) or concordance or (intraclass and correlation*) or discriminative or known group or (factor analysis or factor analyses or factor structure or factor structures) or dimension* or subscale* or (multitrait and scaling and (analysis or analyses)) or item discriminant or interscale correlation* or (error or errors) or individual variability or interval variability or rate variability or (variability and (analysis or values)) or (uncertainty and (measurement or measuring)) or standard error of measurement or sensitiv* or responsive* or (limit and detection) or minimal detectable concentration or interpretab* or ((minimal or minimally or clinical or clinically) and (important or significant or detectable) and (change or difference)) or (small* and (real or detectable) and (change or difference)) or meaningful change or ceiling effect or floor effect or item response model or IRT or rasch or differential item functioning or DIF or computer adaptive testing or item bank or cross-cultural equivalence).mp.

**Search 1: Child and adolescent cohort AND Search 2: Forensic/ secure cohort AND Search 3: Mental illness or Neurodevelopmental disorder AND Search 4: COSMIN filter for measuring properties of measuring instruments** NOT (address.mp. OR biography.mp. OR "case reports".mp. OR comment.mp. OR directory.mp. OR editorial.mp. OR festschrift.mp. OR interview.mp. OR lecture.mp. OR "legal case".mp. OR legislation.mp. OR letter.mp. OR news.mp. OR "newspaper article".mp. OR "patient education handout".mp. OR "popular work".mp. OR congress.mp. OR "consensus development conference".mp. OR "consensus development conference, NIH".mp. OR "practice guideline".mp.)

**CINAHL**

**Search 1: Child and adolescent cohort:**

(MH "Child+") OR (MH "Adolescence")

OR child* or adolesc* or teenage* or juvenile or (young N1 person*) or (young N1 people) or youth*

**Search 2: Forensic/ secure cohort:**

(MH "Forensic Psychiatry+")

OR ((secure OR forensic) n2 (unit OR ward Or hospital Or department Or service* Or psych* OR team)

**Search 3: Mental illness or Neurodevelopmental disorder:**

(MH "Mental Health")

OR ("acute stress" or "adjustment disorder*" or ADNOS or "affective disorder*" or agoraphobi* or phobi* or anxiety disorder? or GAD or (combat n1 (disorder* or fatigue or neuros* or syndrome*)) or compulsi* or (obsessive n1 disorder*) or OCD or depressed or depression or depressive? or dysphori* or dysthymi* or melanchol* or "emotional trauma" or fear or "health anxiety" or hysteri* or MDD or mental* or mood? or neurastheni* or neurotic or neuros* or panic or ((post-trauma* or posttrauma* or "post trauma") n1 stress*) or flashback* or (trauma* n1 (nightmare* or stress or neuros*)) or (psych* n1 (stress or trauma*)) or psychotrauma* or ((sever* or serious* or major* or chronic* or complex* or critical* or endur* or persist* or resist* or acute) n1 (anxiety or psycholog*)) or psychiatr* or psychopathol* or PTSD)

OR ( "schizophrenia spectrum and other psychotic disorder" ) OR "psychotic disorder*" OR psychosis OR schizophreni* OR schizoaffective OR schizophreniform OR psychoses

OR TI executive n1 function* OR AB executive n1 function*

OR (MH "Executive Function")

OR (MH "Language Disorders+")

OR (MH "Memory+")

OR TI dyspraxia OR AB dyspraxia

OR (MH "Apraxia+")

OR TI "attention deficit" OR AB "attention deficit"

OR (MH "Attention Deficit Hyperactivity Disorder")

OR TI autis* OR AB autis*

OR (MH "Autistic Disorder")

OR TI child n1 development OR AB child n1 development

OR (MH "Child Development")

OR TI mental* n1 retard* OR AB mental* n1 retard*

OR TI neurodevelopment OR AB neurodevelopment

OR TI intellectual n1 ability OR intellectual n1 ability

OR TI iq OR AB iq

OR intellectual* n1 impair*

OR (MH "Intellectual Disability")

OR (MH "Fetal Alcohol Syndrome")

OR "fetal alcohol syndrome" OR "foetal alcohol syndrome" OR "fetal alcohol" OR "foetal alcohol" OR fasd OR fae OR arbd OR arnd OR "prenatal alcohol exposure" OR pae OR "alcohol exposed pregnancy" OR aep

**Search 4: COSMIN filter for measuring properties of measuring instruments:**

(MH "Research Instruments") OR (MH "Study Methods")

OR (MH "Psychometrics")

OR TI psychometr* OR AB psychometr*

OR clinimetr* OR clinometr*

OR (MH "Outcomes (Health Care)+")

OR TI "outcome assessment" OR AB "outcome assessment"

OR "outcome measure*"

OR (MH "Nonparticipant Observation")

OR TI "observer variation" OR AB "observer variation"

OR (MH "Health Status Indicators+")

OR (MH "Reproducibility of Results")

OR TI reproducib* OR AB reproducib*

OR (MH "Discriminant Analysis")

OR TI reliab* OR AB reliab*

OR TI unreliab* OR AB unreliab*

OR TI valid* OR AB valid*

OR TI "internal consistency" OR AB "internal consistency"

OR TI homogeneous OR AB homogeneous

OR TI homogeneity OR AB homogeneity

OR TI coefficient OR AB coefficient

OR TI "cross-cultural equivalence" OR AB "cross-cultural equivalence"

OR TI "item bank" OR AB "item bank"

OR TI "computer adaptive testing" OR AB "computer adaptive testing"

OR TI dif OR AB dif

OR TI "differential item functioning" OR AB "differential item functioning"

OR TI rasch OR AB rasch

OR TI irt OR AB irt

OR TI "item response model" OR AB "item response model"

OR TI "floor effect" OR AB "floor effect"

OR TI "ceiling effect" OR AB "ceiling effect"

OR TI "meaningful change" OR AB "meaningful change"

OR ((TI small* OR AB small*) AND ((TI real OR AB real) OR (TI detectable OR AB detectable)) AND ((TI change OR AB change) OR (TI difference OR AB difference)))

OR (((TI minimal OR AB minimal) OR (TI minimally OR AB minimally) OR (TI clinical OR AB clinical) OR (TI clinically OR AB clinically)) AND ((TI important OR AB important) OR (TI significant OR AB significant) OR (TI detectable OR AB detectable)) AND ((TI change OR AB change) OR (TI difference OR AB difference)))

OR TI interpretab* OR AB interpretab*

OR TI "minimal detectable concentration" OR AB "minimal detectable concentration"

OR ((TI limit OR AB limit) AND (TI detection OR AB detection))

OR TI responsiv* OR AB responsiv*

OR TI sensitiv* OR AB sensitiv*

OR TI "standard error of measurement" OR AB "standard error of measurement"

OR ((TI uncertainty OR AB uncertainty) AND ((TI measurement OR AB measurement) OR (TI measuring OR AB measuring)))

OR ((TI variability OR AB variability) AND ((TI analysis OR AB analysis) OR (TI values OR AB values)))

OR TI "rate variability" OR AB "rate variability"

OR TI "interval variability" OR AB "interval variability"

OR TI "individual variability" OR AB "individual variability"

OR TI ( error OR errors ) OR AB ( error OR errors )

OR TI "interscale correlation*" OR AB "interscale correlation*"

OR TI "item discriminant" OR AB "item discriminant"

OR ((TI multitrait OR AB multitrait) AND (TI scaling OR AB scaling) AND ((TI analysis OR AB analysis) OR (TI analyses OR AB analyses)))

OR TI subscale OR AB subscale

OR TI dimension OR AB dimension

OR ((TI "factor analysis" OR AB "factor analysis") OR (TI "factor analyses" OR AB "factor analyses") OR (TI "factor structure" OR AB "factor structure") OR (TI "factor structures" OR AB "factor structures"))

OR TI "known group" OR AB "known group"

OR TI discriminative OR AB discriminative

OR ((TI intraclass OR AB intraclass) AND (TI correlation* OR AB correlation*))

OR TI concordance OR AB concordance

OR TI generali?a* OR AB generali?a*

OR ((replicab* OR repeated) AND (measure OR measures OR findings OR result OR results OR test OR tests))

OR repeatab*

OR TI ( kappa OR kappas ) OR AB ( kappa OR kappas )

OR ((TI interparticipant OR AB interparticipant) OR (TI inter-participant OR AB inter-participant) OR (TI intraparticipant OR AB intraparticipant) OR (TI intra-participant OR AB intra-participant))

OR ((TI interindividual OR AB interindividual) OR (TI inter-individual OR AB inter-individual) OR (TI intraindividual OR AB intraindividual) OR (TI intra-individual OR AB intra-individual))

OR ((TI interassay OR AB interassay) OR (TI inter-assay OR AB inter-assay) OR (TI intraassay OR AB intraassay) OR (TI intra-assay OR AB intra-assay))

OR ((TI interexaminer OR AB interexaminer) OR (TI inter-examiner OR AB inter-examiner) OR (TI intraexaminer OR AB intraexaminer) OR (TI intra-examiner OR AB intra-examiner))

OR ((TI intertechnician OR AB intertechnician) OR (TI inter-technician OR AB inter-technician) OR (TI intratechnician OR AB intratechnician) OR (TI intra-technician OR AB intra-technician))

**Search 1: Child and adolescent cohort AND Search 2: Forensic/ secure cohort AND Search 3: Mental illness or Neurodevelopmental disorder AND Search 4: COSMIN filter for measuring properties of measuring instruments NOT** ((PT address) OR (PT biography) OR (PT "case reports") OR (PT comment) OR (PT directory) OR (PT editorial) OR (PT festschrift) OR (PT interview) OR (PT lecture) OR (PT "legal case") OR (PT legislation) OR (PT letter) OR (PT news) OR (PT "newspaper article") OR (PT "patient education handout") OR (PT "popular work") OR (PT congress) OR (PT "consensus development conference") OR (PT "consensus development conference, NIH") OR (PT "practice guideline"))

**Web of Science Core Collection**

**Search 1: Child and adolescent cohort:**

**TS=**((child* or adolesc* or teenage* or juvenile or (young NEAR/0 person*) or (young NEAR/0 people) or youth*))

**Search 2: Forensic/ secure cohort:**

**TS= (((secure OR forensic ) NEAR/2 (unit OR ward OR hospital OR department OR service* OR psych* OR team ))) AND TS=(((secure OR forensic NEAR/3 (unit OR ward OR hospital OR department OR service* OR psych* OR team ))))**

**Search 3: Mental illness or Neurodevelopmental disorder:**

OR TS= ((affective OR emotional* OR mental* OR psychological*) NEAR/2 (disease$ OR disorder$ OR health* OR ill* OR well* OR stress* OR distress* OR trauma*)) OR "mental disease" OR "mental disorders" OR "mental health" OR anxiety OR "anxiety disorder" OR depression OR "agitated depression" OR "atypical depression" OR "chronic depression" OR dysphoria OR dysthymia OR "endogenous depression" OR "involutional depression" OR "major depression" OR melancholia OR "minor depression" OR "organic depression" OR "postoperative depression" OR "reactive depression" OR "recurrent brief depression" OR "subsyndromal depression"

OR TS= ("treatment resistant depression" OR neurosis OR "affective neurosis" OR "anxiety neurosis" OR hysteria OR neurasthenia OR psychasthenia OR "adjustment disorder" OR "anxiety disorder" OR "acute stress disorder" OR "distress syndrome" OR "generalized anxiety disorder" OR panic OR "posttraumatic stress disorder" OR "separation anxiety" OR "obsessive compulsive disorder" OR compulsion OR obsession OR phobia OR agoraphobia OR claustrophobia OR neophobia OR "social phobia" OR "masked depression" OR "mood disorder" OR "major affective disorder" OR "minor affective disorder" OR "emotional disorder" OR "mood disorders" OR depression OR "depressive disorder" OR "depressive disorder, major" OR "depressive disorder, treatment-resistant" OR "dysthymic disorder" OR "anxiety disorders" OR agoraphobia OR "anxiety, separation" OR "neurocirculatory asthenia" OR "neurotic disorders" OR "obsessive-compulsive disorder" OR "hoarding disorder" OR "panic disorder" OR "phobic disorders" OR "phobia, social" OR "trauma and stressor related disorders" OR "adjustment disorders" OR "stress disorders, traumatic" OR "combat disorders" OR "psychological trauma" OR "stress disorders, post-traumatic" OR "stress disorders, traumatic, acute" OR ("acute stress" OR "adjustment disorder*" OR ADNOS OR "affective disorder*" OR agoraphobi* OR phobi* OR "anxiety disorder$") OR GAD OR (combat NEAR/0 (disorder* OR fatigue OR neuros* OR syndrome*)) OR compulsi* OR (obsessive NEAR/2 disorder*) OR OCD OR depressed OR depression OR depressive$ OR dysphori* OR dysthymi* OR melanchol* OR "emotional trauma" OR fear OR "health anxiety" OR hysteri* OR MDD OR mental* OR mood$ OR neurastheni* OR neurotic OR neuros* OR panic OR ((post-trauma* OR posttrauma*)))

TS= ("Schizophrenia Spectrum and Other Psychotic Disorder*" OR "psychotic disorder*" OR "ACUTE PSYCHOSIS" OR "CHRONIC PSYCHOSIS" OR PSYCHOSIS OR psychosis Schizophrenia OR schizophreni* "SCHIZOAFFECTIVE DISORDER" OR schizoaffective "SCHIZOPHRENIFORM DISORDER" OR schizophreniform "Reactive Psychosis" OR "reactive psychosis" psychoses "reactive psychoses"

OR TS= (psychiatr* OR psychopathol* OR PTSD) OR "Intellectual Disability" OR (intellectual? NEAR/0 impair?) OR IQ OR (intellectual NEAR/0 ability) OR neurodevelopment OR "Child Development" OR "child development" OR "Autistic Disorder" OR (autism OR autistic) OR "Attention Deficit Disorder with Hyperactivity" OR "attention deficit" OR Apraxias OR dyspraxia OR Memory OR memory OR "Language Disorders" OR "Executive Function" OR "executive function?"))

OR TS= (fetal alcohol syndrome OR foetal alcohol syndrome OR fetal alcohol OR foetal alcohol OR fasd OR fae OR arbd OR arnd OR prenatal alcohol exposure OR pae OR alcohol exposed pregnancy OR aep)

**Search 4: COSMIN filter for measuring properties of measuring instruments:**

TS=(instrumentation OR methods) OR TS=(“validation study” OR “comparative study”) OR TS=(Psychometrics) OR (TI=(psychometr*)) OR AB=(psychometr*) OR TS=(clinimetr* OR clinometr*)

OR (TI=(“outcome assessment”)) OR AB=(“outcome assessment”)

OR TS=(“outcome measure*”)

OR TS=(“Observer Variation”)

OR TI=(“observer variation”) OR AB=(“observer variation”)

OR TS=(“Health Status Indicators”)

OR TS=(“Reproducibility of Results”)

OR (TI=(reproducib*)) OR AB=(reproducib*)

OR TS=(“Discriminant Analysis”)

OR (TI=((reliab* OR unreliab* OR valid* OR “coefficient of variation” OR coefficient OR homogeneity OR homogeneous OR “internal consistency”))) OR AB=((reliab* OR unreliab* OR valid* OR “coefficient of variation” OR coefficient OR homogeneity OR homogeneous OR “internal consistency”))

OR (TI=(( cronbach* AND (alpha OR alphas)))) OR AB=(( cronbach* AND (alpha OR alphas)))

OR (TI=((item AND (correlation* OR selection* OR reduction*)))) OR AB=((item AND (correlation* OR selection* OR reduction*)))

OR TS=(agreement)

OR TS=(precision)

OR TS=(imprecision)

OR TS=(“precise values”)

OR (TI=(test-retest)) OR AB=(test-retest)

OR (TI=((test AND retest))) OR AB=((test AND retest))

OR (TI=((reliab* AND (test OR retest)))) OR AB=((reliab* AND (test OR retest)))

OR (TI=(stability)) OR AB=(stability)

OR (TI=((interrater OR inter-rater OR intrarater OR intra-rater))) OR AB=((interrater OR inter-rater OR intrarater OR intra-rater))

OR (TI=((intertester OR inter-tester OR intratester OR intra-tester))) OR AB=((intertester OR inter-tester OR intratester OR intra-tester))

OR (TI=((interobserver OR inter-observer OR intraobserver OR intra-observer))) OR AB=((interobserver OR inter-observer OR intraobserver OR intra-observer))

OR (TI=((intertechnician OR inter-technician OR intratechnician OR intra-technician))) OR AB=((intertechnician OR inter-technician OR intratechnician OR intra-technician))

OR (TI=((interexaminer OR inter-examiner OR intraexaminer OR intra-examiner))) OR AB=((interexaminer OR inter-examiner OR intraexaminer OR intra-examiner))

OR (TI=((interassay OR inter-assay OR intraassay OR intra-assay))) OR AB=((interassay OR inter-assay OR intraassay OR intra-assay))

OR (TI=((interindividual OR inter-individual OR intraindividual OR intra-individual))) OR AB=((interindividual OR inter-individual OR intraindividual OR intra-individual))

OR (TI=((interparticipant OR inter-participant OR intraparticipant OR intra-participant))) OR AB=((interparticipant OR inter-participant OR intraparticipant OR intra-participant))

OR (TI=(kappa)) OR AB=(kappa)

OR (TI=(kappas)) OR AB=(kappas)

OR TS=(repeatab*)

OR TS=(((replicab* OR repeated) AND (measure OR measures OR findings OR result OR results OR test OR tests)))

OR (TI=((generaliza* OR generalisa*))) OR AB=((generaliza* OR generalisa*))

OR (TI=(concordance)) OR AB=(concordance)

OR (TI=((intraclass AND correlation*))) OR AB=((intraclass AND correlation*))

OR (TI=(discriminative)) OR AB=(discriminative)

OR (TI=(“known group”)) OR AB=(“known group”)

OR (TI=((“factor analysis” OR “factor analyses” OR “factor structure” OR “factor structures”))) OR AB=((“factor analysis” OR “factor analyses” OR “factor structure” OR “factor structures”))

OR (TI=(dimension*)) OR AB=(dimension*)

OR (TI=(subscale*)) OR AB=(subscale*)

OR (TI=((multitrait AND scaling AND (analysis OR analyses)))) OR AB=((multitrait AND scaling AND (analysis OR analyses)))

OR (TI=(“item discriminant”)) OR AB=(“item discriminant”)

OR (TI=(“interscale correlation*”)) OR AB=(“interscale correlation*”)

OR (TI=((error OR errors))) OR AB=((error OR errors))

OR (TI=(“individual variability”)) OR AB=(“individual variability”)

OR (TI=(“interval variability”)) OR AB=(“interval variability”)

OR (TI=(“rate variability”)) OR AB=(“rate variability”)

OR (TI=((variability AND (analysis OR values)))) OR AB=((variability AND (analysis OR values)))

OR (TI=((uncertainty AND (measurement OR measuring)))) OR AB=((uncertainty AND (measurement OR measuring)))

OR (TI=(“standard error of measurement”)) OR AB=(“standard error of measurement”)

OR (TI=(sensitiv*)) OR AB=(sensitiv*)

OR (TI=(responsive*)) OR AB=(responsive*)

OR (TI=((limit AND detection))) OR AB=((limit AND detection))

OR (TI=(“minimal detectable concentration”)) OR AB=(“minimal detectable concentration”)

OR (TI=(interpretab*)) OR AB=(interpretab*)

OR (TI=(((minimal OR minimally OR clinical OR clinically) AND (important OR significant OR detectable) AND (change OR difference)))) OR AB=(((minimal OR minimally OR clinical OR clinically) AND (important OR significant OR detectable) AND (change OR difference)))

OR (TI=((small* AND (real OR detectable) AND (change OR difference)))) OR AB=((small* AND (real OR detectable) AND (change OR difference)))

OR (TI=(“meaningful change”)) OR AB=(“meaningful change”)

OR (TI=(“ceiling effect”)) OR AB=(“ceiling effect”)

OR (TI=(“floor effect”)) OR AB=(“floor effect”)

OR (TI=(“item response model”)) OR AB=(“item response model”)

OR (TI=(IRT)) OR AB=(IRT)

OR (TI=(rasch)) OR AB=(rasch)

OR (TI=(“differential item functioning”)) OR AB=(“differential item functioning”)

OR (TI=(DIF)) OR AB=(DIF)

OR (TI=(“computer adaptive testing”)) OR AB=(“computer adaptive testing”)

OR (TI=(“item bank”)) OR AB=(“item bank”)

OR (TI=(“cross-cultural equivalence”)) OR AB=(“cross-cultural equivalence”)

**Search 1: Child and adolescent cohort AND Search 2: Forensic/ secure cohort AND Search 3: Mental illness or Neurodevelopmental disorder AND Search 4: COSMIN filter for measuring properties of measuring instruments NOT** TS=((address OR biography OR “case reports” OR comment OR directory OR editorial OR festschrift OR interview OR lecture OR “legal case” OR legislation OR letter OR news OR “newspaper article” OR “patient education handout” OR “popular work” OR congress OR “consensus development conference” OR “consensus development conference, NIH” OR “practice guideline”))

**Cochrane Database**

**Search 1: Child and adolescent cohort:**

MeSH descriptor: [Adolescent] explode all trees

OR MeSH descriptor: [Child] explode all trees

OR (child* or adolesc* or teenage* or juvenile or (young near/0 person*) or (young near/0 people) or youth*):ti,ab,kw

**Search 2: Forensic/ secure cohort:**

MeSH descriptor: [Forensic Psychiatry] explode all trees

OR ((secure OR forensic ) near/2 (unit OR ward OR hospital OR department OR service* OR psych* OR team )):ti,ab,kw

**Search 3: Mental illness or Neurodevelopmental disorder:**

MeSH descriptor: [Mental Disorders] explode all trees

OR MeSH descriptor: [Depression] explode all trees

OR MeSH descriptor: [Anxiety] explode all trees

OR MeSH descriptor: [Mental Health] explode all trees

OR MeSH descriptor: [Intellectual Disability] explode all trees

OR MeSH descriptor: [Apraxias] explode all trees

OR MeSH descriptor: [Language Disorders] explode all trees

OR ((affective OR emotional* OR mental* OR psychological* ) near/1 (disease? OR disorder? OR health* OR ill* OR well* OR stress* OR distress* OR trauma* )):ti,ab,kw

OR (((acute next stress OR adjustment next disorder OR ADNOS OR affective next disorder* OR agoraphobi* OR phobi* OR anxiety next disorder OR GAD OR (combat near/1 (disorder* OR fatigue OR neuros* OR syndrome* )) OR compulsi* OR (obsessive near/1 disorder* ) OR OCD OR depressed OR depression OR depressive? OR dysphori* OR dysthymi* OR melanchol* OR "emotional trauma" OR fear OR "health anxiety" OR hysteri* OR MDD OR mental* OR mood? OR neurastheni* OR neurotic OR neuros* OR panic OR ((post-trauma* OR posttrauma* OR "post trauma" ) near/1 stress* ) OR flashback* OR (trauma* NEXT (nightmare* OR stress OR neuros* )) OR (psych* NEXT (stress OR trauma* )) OR psychotrauma* OR ((sever* OR serious* OR major* OR chronic* OR complex* OR critical* OR endur* OR persist* OR resist* OR acute ) near/1 (anxiety OR psycholog* )) OR psychiatr* OR psychopathol* OR PTSD))):ti,ab,kw

OR (psychotic disorder* or schizophreni* or schizoaffective or schizophreniform or reactive psychosis OR psychoses OR reactive psychoses):ti,ab,kw

OR ((intellectual* near/1 impair* ) OR IQOR (intellectual near/1 ability ) OR neurodevelopment OR (mental* near/1 retard* ) OR "child development" OR autism OR autistic OR "attention deficit" OR dyspraxia OR memory OR executive near/1 function*):ti,ab,kw

OR (fetal alcohol syndrome OR foetal alcohol syndrome OR fetal alcohol OR foetal alcohol OR fasd OR fae OR arbd OR arnd OR prenatal alcohol exposure OR pae OR alcohol exposed pregnancy OR aep):ti,ab,kw

**Search 4: COSMIN filter for measuring properties of measuring instruments:**

MeSH descriptor: [Outcome Assessment, Health Care] explode all trees

OR MeSH descriptor: [Outcome and Process Assessment, Health Care] explode all trees

OR MeSH descriptor: [Study Characteristics] explode all trees

OR MeSH descriptor: [Observer Variation] explode all trees

OR MeSH descriptor: [Psychometrics] explode all trees

OR MeSH descriptor: [Observer Variation] explode all trees

OR MeSH descriptor: [Health Status Indicators] explode all trees

OR MeSH descriptor: [Reproducibility of Results] explode all trees

OR MeSH descriptor: [Discriminant Analysis] explode all trees

OR ((instrumentation or methods) or (validation study or comparative study) OR (clinimetr* or clinometr*) OR outcome measure* OR agreement OR precision OR imprecision OR precise values OR repeatab* OR ((replicab* or repeated) and (measure or measures or findings or result or results or test or tests))):ti,ab,kw

OR (((outcome assessment OR observer variation OR reproducib* OR (reliab* or unreliab* or valid* or coefficient of variation or coefficient or homogeneity or homogeneous or internal consistency) OR (cronbach* and (alpha or alphas)) OR (item and (correlation* or selection* or reduction*)) OR test-retest OR (test and retest) OR (reliab* and (test or retest)) OR stability OR (interrater or inter-rater or intrarater or intra-rater) OR (intertester or inter-tester or intratester or intra-tester) OR (interobserver or inter-observer or intraobserver or intra-observer) OR (intertechnician or inter-technician or intratechnician or intra-technician) OR (interexaminer or inter-examiner or intraexaminer or intra-examiner) OR (interassay or inter-assay or intraassay or intra-assay) OR (interindividual or inter-individual or intraindividual or intra-individual) OR (interparticipant or inter-participant or intraparticipant or intra-participant) OR kappa OR kappas OR (generaliza* or generalisa*) OR concordance OR (intraclass and correlation*) OR discriminative OR known group OR (factor analysis or factor analyses or factor structure or factor structures) OR dimension* OR subscale* OR (multitrait and scaling and (analysis or analyses)) OR item discriminant OR interscale correlation* OR (error or errors) OR individual variability OR interval variability OR rate variability OR (variability and (analysis or values)) OR (uncertainty and (measurement or measuring)) OR standard error of measurement OR sensitiv* OR responsive* OR (limit and detection) OR minimal detectable concentration OR interpretab* OR ((minimal or minimally or clinical or clinically) and (important or significant or detectable) and (change or difference)) OR (small* and (real or detectable) and (change or difference)) OR meaningful change OR ceiling effect OR floor effect OR item response model OR IRT OR rasch OR differential item functioning OR DIF OR computer adaptive testing OR item bank OR cross-cultural equivalence))):ti,ab

**Search 1: Child and adolescent cohort AND Search 2: Forensic/ secure cohort AND Search 3: Mental illness or Neurodevelopmental disorder AND Search 4: COSMIN filter for measuring properties of measuring instruments**
